# Biocontrol potential of *Chitinophaga flava* HK235 producing antifungal-related peptide chitinocin

**DOI:** 10.3389/fmicb.2023.1170673

**Published:** 2023-05-22

**Authors:** Da Yeon Kim, Jae Woo Han, Jin Woo Lee, Bomin Kim, Yeong Seok Kim, Heung-Tae Kim, Gyung Ja Choi, Hun Kim

**Affiliations:** ^1^Center for Eco-Friendly New Materials, Korea Research Institute of Chemical Technology, Daejeon, South Korea; ^2^Department of Plant Medicine, Chungbuk National University, Cheongju, South Korea; ^3^Department of Medicinal Chemistry and Pharmacology, University of Science and Technology, Daejeon, South Korea

**Keywords:** *Chitinophaga flava*, plant pathogen, biocontrol, antimicrobial peptide, antifungal activity

## Abstract

*Botrytis cinerea* is a necrotrophic fungal pathogen with an extremely broad host range, causing significant economic losses in agricultural production. In this study, we discovered a culture filtrate of bacterial strain HK235, which was identified as *Chitinophaga flava*, exhibiting high levels of antifungal activity against *B. cinerea*. From the HK235 culture filtrate, we isolated a new antimicrobial peptide molecule designated as chitinocin based on activity-guided fractionation followed by characterization of the amino acid composition and spectroscopic analyses. The HK235 culture filtrate and chitinocin completely inhibited both conidial germination and mycelial growth of *B. cinerea* at a concentration of 20% and 200 μg/mL, respectively. In addition to antibiosis against *B. cinerea*, the active compound chitinocin had a broad antifungal and antibacterial activity *in vitro.* When tomato plants were treated with the culture filtrate and chitinocin, the treatment strongly reduced the development of gray mold disease in a concentration-dependent manner compared to the untreated control. Here, considering the potent antifungal property *in vitro* and *in vivo*, we present the biocontrol potential of *C. flava* HK235 for the first time.

## Introduction

*Botrytis cinerea* Pers. is a necrotrophic fungus causing the gray mold disease on over 200 plant species, which results in significant economic losses in agricultural production (Dean et al., 2012; Fillinger and Elad, 2016). Infection and serious damage by this fungus have been observed starting from the seedling stages until post-harvest storage, transport, and retail (Choquer et al., 2007; Droby and Lichter, 2007). To control gray mold disease, the application of chemical fungicides has been considered as one of the most effective and consistent methods (Fillinger and Elad, 2016). However, fungicide-resistant *B. cinerea* strains have been detected in the field after long-term continuous application of fungicides. Thus, gray mold disease is becoming increasingly difficult to control with fungicides (Leroux et al., 2002; Hahn, 2014). Moreover, disease control strategies relying on synthetic fungicides are raising public concern about the harmful impacts on humans and the environment (Abbey et al., 2019). Strict regulatory policies on fungicides for food safety further limit the available chemicals to control gray molds (Rico et al., 2019).

To complement chemical fungicides, the application of microorganisms and/or microbial metabolites has been considered an attractive method to control gray mold disease (Wang et al., 2018; Abbey et al., 2019). In recent decades, many studies have shown that antagonistic bacteria inhibit the growth of *B. cinerea in vitro* by producing antibiotic secondary metabolites and extracellular hydrolytic enzymes (Someya et al., 2001; Haidar et al., 2016). However, only a limited number of antagonistic bacteria and their metabolites are commercially available for the control of gray mold disease in the pre- and post-harvest seasons (Collinge et al., 2022).

To find valuable bacterial biocontrol agents (BCAs), most research efforts have focused on the antibiosis of beneficial microbes at initial screening procedures (Köhl et al., 2019). Currently, the microbial strains from genera *Bacillus*, *Pseudomonas*, and *Agrobacterium* have the most important role in biocontrol, which have been commercialized as a biopesticide. This study showed that a soil-borne bacterium *Chitinophaga flava* HK235 exhibits a promising antifungal activity against *B. cinerea*. Since the genus *Chitinophaga* was first described in 1981, currently, 53 *Chitinophaga* species have been identified with standing in the nomenclature (Parte, 2018).^1^ Bacteria belonging to the genus *Chitinophaga* have been isolated from different environmental sources including the soil, roots, plant rhizosphere, and freshwater sediment (Wang et al., 2014; Li et al., 2017; Tran et al., 2020). *Chitinophaga* spp. have been reported to be aerobic, gram-negative, rod-shaped, non-motile or motile by gliding, yellow pigmented, and capable of hydrolyzing chitin or cellobiose (Kämpfer et al., 2006; Glavina Del et al., 2010). In particular, several *Chitinophaga* spp. strains exhibited antagonistic properties against plant pathogens. For example, *C. einensis* YC6729 exhibited an antifungal activity against *Colletotrichum coccodes, Pythium ultimum*, and *Fusarium moniliforme* (Yasir et al., 2011). A *Chitinophaga* sp. S167 strain producing extracellular chitinases exhibited a disease control efficacy against *Fusarium oxysporum* on tomatoes and nematicidal activities against second-stage juveniles of the root-knot nematode *Meloidogyne incognita* (Sharma et al., 2020). In addition to the ability of *Chitinophaga* spp. to secrete enzymes, it has been reported that *Chitinophaga* spp. produces antimicrobial secondary metabolites such as elansolids and pinensins (Steinmetz et al., 2011; Mohr et al., 2015). However, these secondary metabolites of *Chitinophaga* spp. have not been extensively studied for their potential as biocontrol agents in agriculture.

This study aimed to (1) find a valuable BCA with high antagonistic activity against gray mold disease caused by *B. cinerea* and (2) examine the active metabolites from the selected BCA by chromatographic and spectroscopic methods. To provide information on its biocontrol potential, we investigated the *in vivo* antifungal activity of the BCA and its purified compound. Taken together, our results could provide valuable information to develop new biological control agents for gray mold disease.

## Materials and methods

### Microbial strains and culture conditions

A soil-borne bacterium HK235 was isolated from the rhizosphere soil of tomato plants and deposited at the Korean Agricultural Culture Collection (KACC) under accession no. KACC 81044BP. The isolated bacterial strain was maintained on tryptic soy agar (TSA; BD Difco, Sparks, MD). Additionally, tryptic soy broth (TSB), Luria-Bertani broth (LB), malt extract broth (MB), and nutrient broth (NB) purchased from BD Difco were used for the liquid culture. The HK235 cells were suspended in 20% glycerol solution and kept at –20^°^C until further use. For the *in vitro* antifungal activity assay, *B. cinerea* NY76 (accession no. KACC 48736) provided by the KACC was used and maintained on potato dextrose agar (PDA) medium. For the sporulation, this fungal pathogen was grown on a PDA medium at 20^°^C for 6 days under darkness and then incubated under a 14 h light/10 h dark cycle of illumination for 4 days.

### Whole genome analysis of the HK235 strain

The genomic DNA of the HK235 strain grown in TSB at 30^°^C for 48 h was extracted using QIAamp genomic DNA kits (Qiagen, Hilden, Germany), following the manufacturer’s instructions. The purity and concentration of the genomic DNA were determined with a 2,100 Bioanalyzer system (Agilent Technologies, Palo Alto, CA, USA). The whole genome of the HK235 strain was sequenced with a 20 kb SMRTbell library on the RS II sequencing platform (Pacific Biosciences, Menlo Park, CA, USA) using C4 chemistry with eight single-molecule real-time (SMRT) cells at Macrogen (Seoul, South Korea) (Chin et al., 2013). The cleaned reads were assembled *de novo* using Canu v1.7, and the result was polished with error correction by Pilon v1.23 (Pruitt et al., 2012; Seemann, 2014). All tools were run with default parameters unless otherwise specified. For the whole genome analysis, genome annotation was performed with the NCBI Prokaryotic Genome Annotation Pipeline (PGAP) v6.2 (Tatusova et al., 2016). Average nucleotide identities (ANI) with the currently available genome sequences of *Chitinophaga* spp.^2^ were calculated using Oat 0.93 (Lee et al., 2016; Ciufo et al., 2018). The genome sequence of HK235 was deposited in the NCBI database with an accession code CP073766.

### Inhibitory assay for mycelial growth and conidial germination

To examine the *in vitro* antifungal activity against *B. cinerea*, we investigated the mycelial growth and conidial germination of *B. cinerea* by treatment as previously described (Han et al., 2020). Briefly, a mycelial disk (5 mm in diameter) of *B. cinerea* was inoculated onto PDA medium containing different concentrations of culture filtrate (CF) or isolated active compound, and then, the radial growth of *B. cinerea* was investigated at 7 days post-inoculation (dpi). PDA plates containing 1% dimethyl sulfoxide (DMSO) were prepared as a negative control. For the inhibitory effect on the conidial germination of *B. cinerea*, microplate wells containing a conidial suspension (1 × 10^5^ conidia/mL of PDB) were treated with the CF and active compound, and then, after a 10 h incubation at 20^°^C, the number of germinated conidia was counted by microscopic observation from a total of 100 conidia. All experiments were conducted twice with three replicates.

### Isolation of the active compound from HK235 CF

The HK235 strain was inoculated onto 400 mL of TSB medium in 2 L baffled Erlenmeyer flask and then incubated at 25^°^C for 3 days with an agitation of 150 r/min. The resulting culture broth (5 L) was centrifuged at 10,000 × g for 30 min and passed through Whatman No.1 filter paper (Maidstone, United Kingdom). To isolate antimicrobial compounds, the CF was sequentially extracted with the same volume of *n*-hexane, ethyl acetate, and *n*-butanol. Each resulting layer was evaporated to dryness, yielding an *n*-hexane extract (0.1 g), ethyl acetate extract (0.5 g), *n*-butanol extract (6 g), and water extract (113 g). Based on an antifungal activity-guided bioassay against *B. cinerea*, the *n-*butanol extract was suspended in 1 L of water and then applied onto a Diaion HP-20 column (5 × 30 cm; Mitsubishi Chemical, Tokyo, Japan) with stepwise gradient elution of 0, 20, 40, 60, 80, and 100% aqueous acetone. The 80% aqueous acetone fraction (0.7 g) exhibiting the antifungal activity was subjected to reversed-phase flash column chromatography packed with LiChroprep RP-18 (40–63 μm; Merck, Kenilworth, NJ, United States), eluting with methanol at a flow rate of 5 mL/min. An active fraction (538 mg) was further purified with an LC-6AD high performance liquid chromatography system (Shimadzu, Kyoto, Japan) equipped with a Polaris C18-A column (250 × 21.2 mm, 5 μm; Agilent, Santa Clara, CA, United States). The column was eluted at a flow rate of 5 mL/min with 20–80% aqueous acetonitrile (containing 0.1% formic acid) at a linear gradient over a 50 min uninterrupted interval. The effluent was monitored with a SPD-M10Avp photodiode array detector (Shimadzu). Finally, the purified compound (37 mg) was obtained and kept at –20^°^C until further analysis. The purified compound was analyzed with a Waters 515 high-performance liquid chromatography (HPLC) system equipped with a Phenomenex Luna C18(2) column (4.6 × 250 mm, 5 μm). The mobile phase consisted of solvent A (deionized water containing 0.1% formic acid) and solvent B (methanol) using a gradient elution as follows: 20–100% B at 0–15 min and 100% B at 15–30 min. The flow rate was 1 mL/min, and the effluent was monitored at 210 nm.

### Mass spectroscopic analysis

To identify the structure of the isolated compound, mass spectra were acquired with an Autoflex Speed TOF/TOF mass spectrometer (Bruker, Billerica, MA, United States), and 2,5-dihydroxybenzoic acid was used as a matrix. Spectra were obtained by positive ion detection and reflector mode. Monoisotopic masses were detected.

### Amino acid composition analysis

Amino acid composition analysis of the purified compound was performed with the Pico-Tag system (Waters, Milford, MA, United States) at the Korea Basic Science Institute (Daejeon, South Korea). The pure compound was hydrolyzed followed by labeling with phenylisothiocyanate (PITC) according to the Waters Pico-Tag protocol. The PITC-labeled hydrolysates were analyzed by a HPLC system equipped with a Waters Pico-Tag column (3.9 × 300 mm, 4 μm). The mobile phase consisted of solvent A (6% acetonitrile in 140 mM sodium acetate) and solvent B (60% acetonitrile in water) using the following gradient elution: 0–14% B at 0–9 min, 14–20% B at 9–9.2 min, 0–46% B at 9.2–17.5 min, and 46–100% B at 17.5–17.7 min. The flow rate was 1 mL/min, and the effluent was monitored at 254 nm.

### *In vivo* antifungal activity assay

To investigate the disease control efficacy against tomato gray mold, the HK235 CF was prepared at concentrations of 11, 33, and 100% by diluting it in distilled water. A purified compound was prepared by dissolving it in 1% DMSO. Chemical fungicide fludioxonil (50 μg/mL) and 1% DMSO were used as positive and negative controls, respectively. All samples contained 0.025% Tween 20 as a surfactant. Two-leaf stages of tomato seedlings (*Solanum lycopersicum* cv. Seokwang; Hungnong Seeds, Seoul, South Korea) were grown in a greenhouse at 25 ± 5^°^C for 3–4 weeks. The treatments were applied onto the tomato seedlings by a spray method and then air-dried for 24 h. The treated tomato plants were inoculated with a conidial suspension (5 × 10^5^ conidia/mL) of *B. cinerea* by the spray method (Nguyen et al., 2022). The inoculated seedlings were incubated in a humidified chamber at 20^°^C for 3–4 days. The disease control efficacy (%) was calculated based on the lesion as described previously (Ngo et al., 2021).

To evaluate the *in vivo* antifungal activity on fruits, strawberry fruits (*Fragaria* × *ananassa* cv. Seolhyang) were surface-sterilized using 0.01% (v/v) sodium hypochlorite for 1 min and then rinsed with running water. After air-drying, each fruit was punched by a 3 mm-diameter cork-borer and treated with CF and pure compound. The treated fruits were point-inoculated with 5 μL of the *B. cinerea* conidial suspension (1 × 10^6^ conidia/mL) and incubated in a humidified chamber at 20^°^C for 5 days.

For the penetration assay, the onion epidermis was used for microscopic observation of the penetration process of *B. cinerea* in the presence of the HK235 CF or pure compound solutions. After treatment of each sample, the epidermis was inoculated with 20 μL of the *B. cinerea* conidial suspension (1 × 10^5^ conidia/mL) and then incubated in a moist container at 20^°^C for 24 h. The inoculated epidermis was stained with lactophenol cotton blue and photographed.

### Statistical analysis

For the quantitative data of this study, experiments were performed independently at least two times with three biological replicates unless indicated. Data were subjected to one-way ANOVA, and the means of the treatments were separated by Duncan’s multiple range test (*p* < 0.05) using the R-software (Version 4.0.5). All values were expressed as the mean ± standard deviation, and the significant differences (*p* < 0.05) were indicated with different letters.

## Results

### Identification of the HK235 strain showing antifungal activity against *Botrytis cinerea*

**FIGURE 1:**
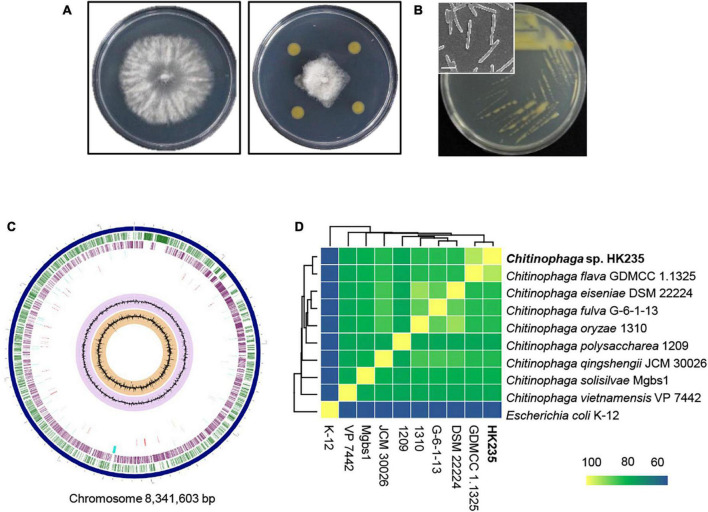
The morphology and phylogenetic analyses of an antagonistic bacterium HK235. **(A)** In dual culture assay, the HK235 strongly inhibited the mycelial growth of *Botrytis cinerea* on potato dextrose agar (PDA). **(B)** A yellowish colony was observed 3 days after incubation on tryptic soy agar (TSA). The bacterial cells were imaged with a field emission scanning electron microscope TESCAN MIRA3 (TESCAN, Brno, Czech Republic). The size bar indicates 2 μm. **(C)** The circularized genome map of *Chitinophaga flava* HK235. From outermost to innermost data: contigs (dark blue); forward coding sequences (green); reverse coding sequences (purple); non-CDS features (sky blue); antimicrobial genes (red); GC content (lilac); GC skew (orange). **(D)** Phylogeny of *Chitinophaga* species based on a heatmap with row and column dendrograms from the average nucleotide identity of genomes of the HK235 and other *Chitinophaga* spp.

During the course of screening for antagonistic bacteria, we found that a HK235 strain exhibited a promising antifungal activity against *B. cinerea* (Figure 1A). The HK235 strain grown on TSA medium was observed to be yellowish, smooth and opaque, and rod-shaped with a size of 4.1–4.3 μm in length and 0.36–0.43 μm in diameter (Figure 1B). Based on the 16S rRNA gene sequence (1,353 bp) of the HK235 strain, phylogenetic analyses revealed that the HK235 strain belongs to the genus *Chitinophaga* and is most closely related to *C. flava* K3CV102501 with 99.48% similarity (Supplementary Figure 1). Furthermore, we found that the whole genome of the HK235 strain contains 6,324 coding DNA regions, 76 tRNAs, 18 rRNAs, 3 ncRNAs, 5 non-coding RNAs, and 18 pseudogenes (Figure 1C). Considering that *Chitinophaga* spp. produces various bioactive metabolites, we analyzed the HK235 whole genome by antiSMASH, PRISM, and RiPPMiner (Skinnider et al., 2017; Agrawal et al., 2021; Blin et al., 2021). The results showed that the HK235 genome contains 25 secondary metabolite biosynthetic gene clusters (BGCs) for 10 non-ribosomal peptides (NRPs), three ribosomally synthesized and post-translationally modified peptides (RiPPs), seven terpenes, and others (aryl polyene, resorcinol, aminoglycoside, and siderophore) (Supplementary Tables 1, 2). With the genome sequences of eight known *Chitinophaga* strains, the ANI dendrogram-heatmap revealed that the HK235 strain was closely related to *C. flava* GDMCC 1.1325 (GenBank accession No. GCA_003308995) with the highest ANI value of 93.24% at the genome level (Figure 1D). Therefore, we identified the HK235 strain as a *Chitinophaga flava.*

### Isolation and identification of antifungal substances of *Chitinophaga flava* HK235

**FIGURE 2:**
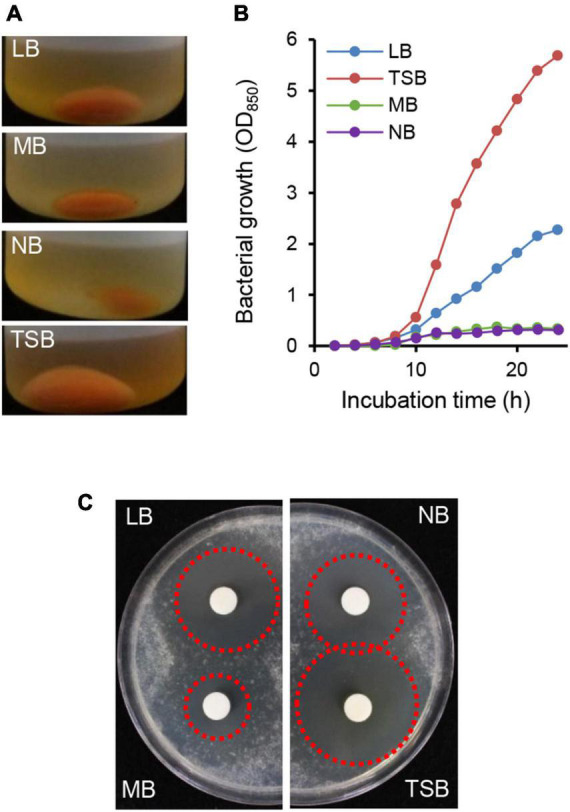
Comparison of bacterial growth and antifungal activity of HK235 grown in four different media. **(A)** The centrifuged cell pellets of HK235 cultures. After 3 days of shaking incubation, HK235 cultures were centrifuged at 10,000 × g for 10 min. **(B)** The bacterial growth of the HK235 strain. The bacterial growth was monitored by optical density (OD_850_) at 2 h intervals. **(C)** Paper disk-agar diffusion assay of culture filtrates against *Botrytis cinerea*. The antifungal activity with a clear zone (red dotted circle) was observed at 3 dpi.

When the HK235 strain was grown in various liquid media such as LB, MB, NB, and TSB, the TSB medium supported a higher yield of cell mass of the HK235 strain (Figure 2). In the paper disk-agar diffusion assay using the HK235 CF derived from each culture medium, the HK235 CF from the TSB medium exhibited the most effective antifungal activity against *B. cinerea* (Figure 2). Therefore, we decided to use 3 days-old HK235 culture grown in TSB medium to isolate the active compound.

**FIGURE 3:**
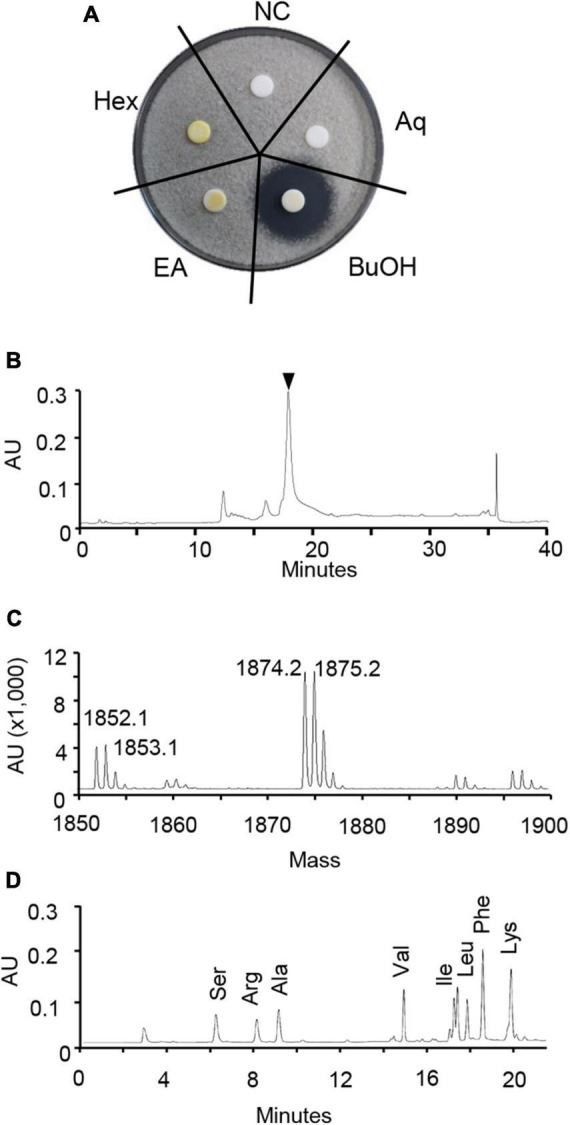
Identification of chitinocin. **(A)** For the paper disk-agar diffusion assay, the partitioned fractions were applied onto a paper disk on potato dextrose agar (PDA) containing a conidial suspension (1 × 10^4^ conidia/mL) of *Botrytis cinerea.* Hex, *n*-hexane; EA, ethyl acetate; BuOH, *n*-butanol; Aq, water layer; NC, non-treatment control. **(B)** High performance liquid chromatograph (HPLC) chromatogram of chitinocin. The black reversed triangle represents a purified chitinocin at a retention time of 17.8 min. **(C)** Positive MALDI-TOF-MS spectrum of chitinocin. **(D)** Amino acid composition analysis of chitinocin.

To isolate the active compound, the HK235 CF was sequentially partitioned with *n*-hexane, ethyl acetate, and *n-*butanol. Of the solvent layers, the *in vitro* antifungal activity was exclusively observed from the *n*-butanol layer at a concentration of 1,000 μg/mL (Figure 3). Therefore, the *n*-butanol layer was purified through a series of column chromatography based on antifungal activity-guided fractionation. The resulting purified compound had a MALDI-TOF mass spectrum showing only one series of [M + H]^+^ and [M + Na]^+^ ions at m/z 1,853 and 1,875, respectively (Figure 3). A doubly charged [M–2H]^2–^ ion at m/z 925 observed in an ESI mass spectrum of the purified compound also supported a molecular weight of 1,852 Da (Supplementary Figure 2). Considering that the compound showed the end absorption in the UV spectrum and a positive ninhydrin reaction, the compound is likely to be a peptide containing free amino groups. When the amino acid composition of the compound was analyzed, we found that it consisted of Ala, Leu, Ile, Val, Phe, Ser, Lys, and Arg (Figure 3). For further identification of the chemical structure, MALDI-TOF/TOF-MS analysis was performed, but we did not obtain a resulting peptide sequence (Supplementary Figure 3). Despite the failure to obtain a peptide sequence, all spectroscopic data including the ^1^H, ^13^C, DEPT, COZY, HMBC, and HSQC NMR data (Supplementary Figures 4–9) were totally different from those previously reported. Although structure elucidation was not successful here, the results of the amino acid composition and spectroscopic analysis suggest that the purified active compound is likely to be a new antifungal molecule. Thus, we named the active compound isolated from the HK235 CF as chitinocin.

### *In vitro* antimicrobial activity of *Chitinophaga flava* HK235 CF and chitinocin

**FIGURE 4:**
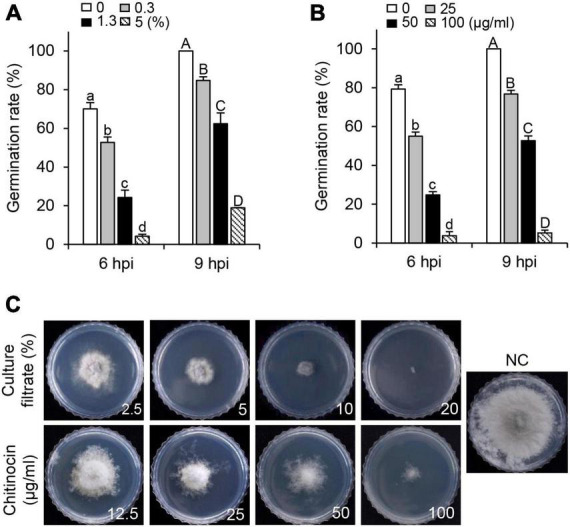
*In vitro* inhibitory effects of the HK235 CF and chitinocin. **(A,B)** Conidial germination inhibition by HK235 CF (0.3, 1.3, and 5%) and chitinocin (25, 50, and 100 μg/mL). The number of germinated conidia was counted from a total of 100 conidia at 6 and 9 hpi. The bars represent the mean ± standard deviation of two runs with three replicates. Different letters indicate significant differences at *p* < 0.05. **(C)** Mycelial growth of *B. cinerea* grown on potato dextrose agar (PDA) medium at 7 dpi. The PDA medium containing 1% dimethyl sulfoxide (DMSO) was used as a negative control (NC).

To examine the *in vitro* antifungal activity of the CF and chitinocin, we investigated the inhibitory effects on conidial germination and mycelial growth of *B. cinerea*. When PDB media were supplemented with CF, the inhibitory activity for conidial germination was observed in a concentration-dependent manner, and a decreased inhibition activity was observed also at 9 hpi compared to the 6 hpi in the treatment group with the same concentration (Figure 4). Similarly, chitinocin with a MIC value of 200 μg/mL exhibited a germination inhibition activity in a concentration-dependent manner (Figure 4). Besides conidial germination inhibition, when PDA media were supplemented with the CF and chitinocin, these treatments inhibited mycelial growth: 20% of the CF and 200 μg/mL of chitinocin completely inhibited mycelial growth (Figure 4 and Supplementary Table 3). To explore the antimicrobial activity spectrum of chitinocin, we measured the MIC values against each of the five phytopathogenic fungi and bacteria. Our results showed that chitinocin has a broad-spectrum antimicrobial activity against *C. coccodes, Phytophthora infestans, Agrobacterium tumefaciens, Burkholderia cepacia, Erwinia amylovora*, and *Ralstonia solanacearum* with MIC values of 50, 12.5, 6.3, 50, 50, and 6.3 μg/mL, respectively (Supplementary Table 3).

### Disease control efficacy against gray mold

**FIGURE 5:**
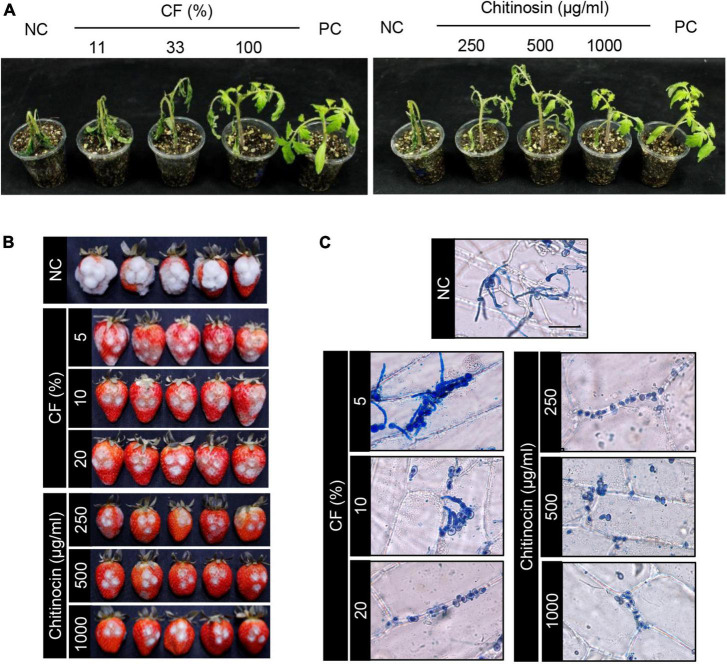
*In vivo* antifungal activity of the HK235 culture filtrate (CF) and chitinocin against gray mold caused by *Botrytis cinerea*. **(A)** Representatives of the plants treated with CF and chitinocin. Treatments of fenhexamid (100 μg/mL) and 1% DMSO were used as positive and negative controls (PC and NC), respectively. Plants were inoculated with a conidial suspension (1 × 10^5^ conidia/mL) of *B. cinerea* at 24 h after treatment with CF and chitinocin. **(B)** Strawberries treated with CF and chitinocin. Five microliter aliquot of conidial suspension (1 × 10^6^ conidia/mL) of *B. cinerea* inoculated after treatment with CF and chitinocin. **(C)** Effect of CF and chitinocin on the penetration process of the *B. cinerea* conidia in onion epidermal cells. The inoculated epidermis was stained with lactophenol cotton blue and photographed at 24 hpi.

To investigate disease control efficacy, we treated the plants with samples that were derived from the HK235 culture prior to the inoculation of the fungal pathogen. After 3 dpi, we found that the 1-fold and 3-fold diluted CFs significantly reduced the disease development of tomato gray mold with control values of 69 and 40%, respectively, compared to the non-treatment control (Figure 5 and Table 1). When chitinocin was applied onto the tomato seedlings, the treatments at a concentration of 250, 500, and 1,000 μg/mL exhibited disease control efficacies with control values of 48, 71, and 92%, respectively (Figure 5 and Table 1). In addition, treatment with the sample from the BuOH extraction layer containing chitinocin also showed a disease control efficacy with control values of 72% at a concentration of 3,000 μg/mL (Table 1). Our results show that all samples reduced in a concentration-dependent manner the development of tomato gray mold. Similarly, we observed that the CF and chitinocin were effective in reducing the gray mold development on mature strawberries. The growth of *B. cinerea* on strawberries was remarkably inhibited by the treatment with the CF (5, 10, and 20%) and chitinocin (250, 500, and 1,000 μg/mL) on the strawberry fruits (Figure 5). Meanwhile, for the non-treatment control, we observed that the *B. cinerea*-infected strawberries were completely covered with the mycelia of *B. cinerea* and subsequently turned a gray color, softened, and rotted within a few days (Figure 5).

**TABLE 1:** *In vivo* antifungal activity against tomato gray mold.

Treatment	Concentration	Disease control (%)^a^
CF	11%	14 ± 0 d
	33%	40 ± 11 bcd
	100%	69 ± 4 ab
BuOH layer	500 μg/mL	26 ± 21 cd
	1,000 μg/mL	50 ± 17 bc
	3,000 μg/mL	72 ± 20 ab
Chitinocin	250 μg/mL	48 ± 17 bc
	500 μg/mL	71 ± 14 ab
	1,000 μg/mL	92 ± 2 a
Fenhexamid	20 μg/mL	94 ± 2 a
	100 μg/mL	100 a

^a^Disease control efficacy (%) represents the mean of three replicates. The values represent the mean ± standard deviation of two runs with three replicates. Different small letters in each column indicate a significant difference at *p* < 0.05 according to Duncan’s multiple range test.

To examine an effect of CF and chitinocin on fungal penetration, onion epidermal cells were treated with the samples prior to inoculation of *B. cinerea* conidial suspension. From the non-treatment control, we observed that conidia and hyphae on the surface were stained with lactophenol cotton blue, and invasively growing hyphae remained colorless also (Figure 5). However, the treatment with 5% CF exhibited few hyphae and conidia on the onion surface and no penetrating hyphae (Figure 5). At a higher concentration of the CF, conidia failed to germinate. Similarly, chitinocin completely inhibited the germination of the *B. cinerea* conidia on the onion epidermal tissues at all the tested concentrations (Figure 5).

## Discussion

In recent decades, there has been continuous exploration of various bacteria possessing antagonistic properties against *B. cinerea* (Köhl et al., 2019; Sellitto et al., 2021). However, only a limited number of antagonistic bacteria and their metabolites have been commercially available as natural fungicides for the control of *Botrytis* gray mold in the pre- and post-harvest seasons (Collinge et al., 2022). In our study, the *C. flava* HK235 exhibited promising *in vitro* and *in vivo* antifungal activity to control *B. cinerea* as well as other plant pathogenic fungi and bacteria. Furthermore, we isolated a pure active compound chitinocin from the *C. flava* HK235 and tried to identify its chemical structure based on its spectroscopic analysis. In this study, we found that chitinocin (M.W. 1,852 Da) exhibited the presence of symmetry with a double of Ala, Leu, Ile, Val, Phe, Ser, Lys, and Arg residues. However, the amino acid sequence of chitinocin could not be determined by MS/MS and 2D NMR analyses because of the MS fragmentation complexity and the low intensity of the NMR signals between the α-carbons and amide protons. Although the structure of chitinocin was not completely identified here, the spectroscopic data of chitinocin were entirely different from those of previously reported peptide antibiotics including several compounds isolated from *Chitinophaga* and the closely related genera *Flexibacter* and *Cytophaga* (Evans and Napier, 1978; Katayama et al., 1985, 1993; Shoji et al., 1988; Yasumuro et al., 1995; Mohr et al., 2015), suggesting that chitinocin could be a novel antibiotic peptide.

In this study, chitinocin exhibited the end absorption in the UV spectrum and a positive ninhydrin reaction, suggesting that the compound is likely to be a peptide containing free amino groups which can be biosynthesized by the NRPS system. Furthermore, considering the molecular weight and amino acid composition of chitinocin, it can be assumed that chitinocin consists of approximately 16-18 amino acid residues. Although the HK235 genome contains a total of 13 BGCs associated with the synthesis of RiPPs and NRPs, their amino acid composition and the number of residues differed from those of chitinocin. Nevertheless, based on the prediction of NRPSs, there are two possible NRPS related to chitinocin. An NRPS with fifteen adenylation domains of a BGC (gene locus of 1703066–1804498) could be responsible for [(X) + (Val, D-Thr, D-Ala, Asp, Phe) + (D-Val, Ser, D-X, X) + (D-Ile, Asp, Leu, Asp, Ile)] (Supplementary Table 1). Another NRPS with nine adenylation domains of a BGC (gene locus of 967071–1036484) could be responsible for [(X, Leu, X, Thr, D-Leu, X) + (D-Phe) + (Val, Asp)] (Supplementary Table 1). However, to comprehensively understand the structure of chitinocin, alternative methods such as X-ray crystallography or computational modeling to complement NMR data are needed.

Bacterial taxa including Chitinophaga are receiving increased attention as novel target genera with high biosynthetic potential (Brinkmann et al., 2022). Currently, two kinds of antibiotics have been reported from *Chitinophaga* spp.: elansolids and pinensins exhibiting promising activity against gram-positive bacteria and fungi, respectively (Steinmetz et al., 2011; Mohr et al., 2015). However, there is limited information on the mode of action of the antibiotics produced by *Chitinophaga* spp. against plant pathogens.

In addition to antibiosis, bacteria producing extracellular cell wall-degrading enzymes such as chitinases, glucanases, and cellulases have been widely demonstrated as inhibiting fungal growth and can be effective in controlling fungal diseases (Quecine et al., 2008; Martínez-Absalón et al., 2014). *Aureobasidium pullulans* producing β-1,3-glucanses and chitinase was reported to be effective in controlling various post-harvest decays on apple and table grape fruits (Castoria et al., 2001). Foliar application of *Serratia plymuthica* producing proteases and chitinases protected cucumber leaves from attacks by *B. cinerea* and *Sclerotinia sclerotiorum* (Kamensky et al., 2003). *Serratia marcescens* exhibits a strong antifungal activity against *B. cinerea in vitro* and produces antibiotic prodigiosin and chitinolytic enzymes that synergistically inhibit conidial germination of *B. cinerea* (Someya et al., 2001). In this study, we observed that the HK235 strain is able to produce the extracellular chitinolytic enzyme (Supplementary Figure 10). The chitinolytic activity along with chitinocin production might be one of the key factors in the development of a biofungicide using the HK235 strain.

Given the increase in resistance to current fungicides, the discovery and agronomical application of antagonistic bacteria with multiple modes of action against plant pathogens are important and worthy of attention (Zhang et al., 2020; Li et al., 2021). A number of antifungal metabolites produced by various bacterial strains of *Bacillus*, *Paenibacillus*, *Brevibacillus*, *Pseudomonas*, *Burkholderia*, and *Streptomyces* have been reported, and their antibiotic peptides have been extensively studied in the context of biological control (Depoorter et al., 2016; Yang and Yousef, 2018; Zhang et al., 2020; Li et al., 2021). Antibiotic peptides have been also highlighted for their potential use in biotechnological and biopharmaceutical applications because of their surfactant properties (Ongena and Jacques, 2008). With a broad antimicrobial activity, in this study, we described the effectiveness of chitinocin for inhibiting the growth of *B. cinerea* and for mitigating fungal infection or contamination on leaves and fruits. Most antimicrobial mechanisms of antibiotic peptides (e.g., gramicidin S and tyrocidine) focus on the change in cell permeability and/or disruption of cell integrity (Buda De Cesare et al., 2020). Similarly, mycelia and conidia of *B. cinerea* treated with chitinocin exhibited increased cell permeability to propidium iodide compared to the untreated control (Supplementary Figure 11). However, given that the target site of peptide antibiotics is not limited to the cell membrane (Rautenbach et al., 2016), further research such as the antimicrobial mechanism of chitinocin is still required to expand our knowledge on peptide structures and their target biomolecules.

## Conclusion

In this study, our results showed that *C. flava* HK235 isolated from the rhizosphere soil of tomato plants exhibit a biocontrol potential for the first time. Although biological activities have been reported from *Chitinophaga* spp., there is limited information on *C. flava* and its secondary metabolites in plant disease control efficacy. Given that *C. flava* HK235 CF effectively controlled tomato gray mold disease caused by *B. cinerea*, we isolated an antibiotic peptide compound from the BuOH extraction layer derived from the HK235 CF. In addition to *B. cinerea*, the *in vitro* antimicrobial assay results revealed the broad antifungal and antibacterial spectrum of chitinocin, including *C. coccodes, P. infestans, E. amylovora*, and *R. solanacearum.* The natural compound chitinocin also exhibited plant disease control efficacies against gray mold disease. Taken together, our results suggest that *C. flava* HK235 and its antimicrobial compound have great potential to be developed as new biocontrol agents for agricultural fields.

## Data availability statement

The datasets presented in this study can be found in online repositories. The names of the repository/repositories and accession number(s) can be found in the article/Supplementary material.

## Author contributions

DK, JH, JL, BK, and YK performed the research and the data analysis. H-TK, GC, and HK designed the research study and supervised the project. DK, JH, and HK contributed to the manuscript preparation. All authors have revised and approved the final version of the manuscript.

## Footnotes

1
https://lpsn.dsmz.de

2
ncbi.nlm.nih.gov
