# Supplementary material for: Biocontrol potential of Chitinophaga flava HK235 producing antifungal-related peptide chitinocin
Source: Front Microbiol. 2023 May 22;14:1170673. doi: 10.3389/fmicb.2023.1170673 (PMC10239826; doi:10.3389/fmicb.2023.1170673)
Supplement: Supplementary file 1 [file Data_Sheet_1.PDF]

Supporting Information

Biocontrol Potential of *Chitinophaga flava* HK235 Producing Antifungal-related Peptide Chitinocin

Da Yeon Kim[1,2†], Jae Woo Han[1†], Jin Woo Lee[1,3], Bomin Kim[1,3], Yeong Seok Kim[1,3], Heung-Tae Kim[2], Gyung Ja Choi[1,3] and Hun Kim[1,3]*

[1]*Center for Eco-friendly New Materials, Korea Research Institute of Chemical Technology, Daejeon, South Korea*

[2]*Department of Plant Medicine, Chungbuk National University, Cheongju, South Korea,*

[3]*Department of Medicinal Chemistry and Pharmacology, University of Science and Technology, South Korea*

[†]These authors contributed equally to this work.

***Correspondence:** Hun Kim hunkim@krict.re.kr

Running title: Biocontrol Potential of *Chitinophaga flava*

Materials and Methods

Broth Microdilution Method for MICs

Minimum inhibitory concentration (MIC) values of the chitinocin were determined against plant pathogenic fungi and bacteria using the 2-fold serial dilution method as previously described (Nguyen et al., 2022). The following plant pathogenic fungi provided by the Korea Agricultural Culture Collection (KACC) were used: *Alternaria brassicicola* (KACC 40036), *Botrytis cinerea* (KACC 48736), *Colletotrichum coccodes* (KACC 48737), *Magnaporthe oryzae* (KACC 46552), and *Phytophthora infestans* (KACC 48738). Bacterial strains *Agrobacterium tumefaciens* SL2434, *Burkholderia cepacia* SL4269, *Clavibacter michiganensis* subsp. *michiganensis* SL4135, *Erwinia amylovora* TS3128, and *Ralstonia solanaceanum* SL1944 were provided by the National Academy of Agricultural Sciences (Wanju, Korea). Potato dextrose agar (BD Difco, Sparks, MD) or oatmeal agar (BD Difco) were used for the growth of plant pathogenic fungi. *B. cinerea* and *P. infestans* were incubated at 20 °C and other fungi at 25 °C for 6–8 days. To induce sporulation of *M. oryzae*, *B. cinerea* and *P. infestans*, they were incubated under a 12-h photoperiod. For the antibacterial assay, a single colony of plant pathogenic bacteria was transferred to a 5 mL tryptic soy broth (BD Difco) in a 15-mL culture tube, followed by incubation for 1–3 days at 30 °C with an agitation of 150 r/min. Fungal spore and bacterial cell suspension were added to the 96-well plates at a final concentration of 1×10^4 cells/mL. Chitinocin (20 mg/mL) dissolved in dimethyl sulfoxide (DMSO) was serially 2-fold diluted at initial concentrations of 200 μg/mL. All treatments contained no more than 1% DMSO (v/v). The 96-well plates were incubated for 2−3 days, and the MIC values were determined by visual inspection of complete growth inhibition. Assays were performed two times with three replicates for each treatment.

Phylogenic Analysis of the HK235 Strain Based on the 16S rRNA

Genomic DNA was prepared from freshly grown HK235 cells using the cetyltrimethylammonium bromide (CTAB) method as previously described (Chen and Ronald, 1999). The 16S rRNA gene was amplified using the universal primers 27F (5'-AGAGTTTGATCMTGGCTCAG-3') and 1492R (5'-TACGGYTACCTTGTTACGACTT-3'). The amplicons were purified using the Expin PCR purification kit (GeneAll, Seoul, Korea) and then sequenced by Macrogen Inc. (Daejeon, Korea). For phylogenetic analysis, the consensus DNA sequence of the 16S rRNA gene was subjected to sequence-similarity searches using BLASTn (https://www.blastn.ncbi.nlm.nih.gov). The sequences of the 16S rRNA gene were aligned using ClustalW implemented in MEGA X (Kumar et al., 2018). The phylogenetic tree was generated using the maximum-likelihood, neighbor-joining, and maximum-evolution methods. The robustness of the inferred trees was evaluated by bootstrapping with 1,000 replications.

MS/MS and NMR Spectroscopic Analyses

Sequence analysis of the purified compound was performed by MALDI-LIFT-TOF/TOF mass spectrometry on a Bruker Autoflex Speed TOF/TOF (Billerica, MA, USA) in collision-

induced dissociation (CID) mode (Vater et al., 2018). The 1D and 2D nuclear magnetic resonance (NMR) spectra were recorded by a Bruker Advance 700 MHz spectrometer (Bruker BioSpin, Rheinstetten, Germany) in DMSO-d_6 (99.9 atom% D, Cambridge Isotope Laboratories, Tewksbury, MA, USA). Chemical shifts were referenced to solvent peaks (δ_H 2.50 ppm and δ_C 39.5 ppm).

Chitinase Activity

The colloidal chitin was prepared to observe the chitinase activity of HK235 (Hsu and Lockwood, 1975; Ramirez et al., 2004). Finely grounded chitin (10 g) was added to 100 mL of 12 N hydrochloric acid with stirring for 1 h, then passed through cheesecloth. Ice-cold distilled water was added and kept at 4 ºC to allow precipitation of colloidal chitin. The gelatinous white material was separated by filtration and washed with distilled water until the filtrate had a pH of 6.5. Ten microliters of an HK235 culture (OD_{600} = 0.6) was point-inoculated onto colloidal chitin agar (20 g of colloidal chitin, 0.7g of K_2HPO_4, 0.3 g of KH_2PO_4, 0.5 g of $MgSO_4 \cdot 5H_2O$, 0.01 g of $FeSO_4 \cdot 7H_2O$, 0.001 g of $ZnSO_4$, 0.001 g of $MnCl_2$, and 20 g of agar in 1 L distilled water; pH 7.0±0.2) and incubated at 25 ºC until halo formation.

Cell Permeability

B. cinerea mycelia and conidia 1/2 PDB liquid medium were treated with chitinocin (200 µg/mL) for 24 h at 20 °C, and then stained with 2 µM propidium iodide. As a negative and positive control, 1% DMSO and 70% ethanol were used, respectively. The treated mycelium and conidia were stained with 2 µM propidium iodide (PI; Sigma, USA) for 1 h at 4°C under light-shielded conditions. After washing the samples with 1 mL of 0.01 M phosphate-buffered saline (pH 7.2) to remove PI, microscopic observation was performed with BS53 10X magnification on an Olympus BS53 microscope using CellSens Dimension 1.6 software (Olympus Corp., Münster, Germany). The used excitation and emission wavelengths were 535 nm and 615 nm, respectively.

References

Chen, D.H., Ronald, P. C., 1999. A rapid DNA minipreparation method suitable for AFLP and other PCR applications. Plant Mol. Biol. Report 17, 53–57

Hsu, S. C., Lockwood, J., 1975. Powdered chitin agar as a selective medium for enumeration of actinomycetes in water and soil. Appl. Microbiol. 29, 422–426.

Kumar, S., Stecher, G., Li, M., Knyaz, C., Tamura, K., 2018. MEGA X: molecular evolutionary genetics analysis across computing platforms. Mol. Biol. Evol. 35, 1547.

Nguyen, M. V., Han, J. W., Kim, H., Choi, G. J., 2022. Phenyl ethers from the marine-derived fungus *Aspergillus tabacinus* and their antimicrobial activity against plant pathogenic fungi and bacteria. ACS Omega 7, 33273–33279.

Ramırez, M. G., Avelizapa, L. R., Avelizapa, N. R., Camarillo, R. C., 2004. Colloidal chitin stained with Remazol Brilliant Blue R®, a useful substrate to select chitinolytic microorganisms and to evaluate chitinases. J. Microbiol. Methods 56, 213–219.

Vater, J., Herfort, S., Doellinger, J., Weydmann, M., Borriss, R., Lasch, P., 2018. Genome mining of the lipopeptide biosynthesis of *Paenibacillus polymyxa* E681 in combination with mass spectrometry: discovery of the lipoheptapeptide paenilipoheptin. ChemBioChem 19, 744–753.

Table S1. Secondary metabolite biosynthetic gene clusters (BGCs)[a] in the HK235 genome.

Region	Type	Gene locus	Most similar known gene cluster	Similarity (%)	NRPS prediction
1	NRPS–T1PKS hybrid	77774–217821			(mal, Ala) + (Ala, Asp, Asp, Ala, Ala, Ala, X) + (X) + (Thr, D-Ile, X) + (Ala, X) + (D, X, Asn, X) + (D-Ser, Asn, Tyr) + (X) + (Gly)
2	NRPS	237182–399046			(D-Leu, X, Ile, Ile, D-Thr, Val) + (Val, Val, Thr, D-Leu, Ile, Asn, D-Thr, X, Leu, Asn, D-Asn, D-Ile) + (D-Leu, X, D-Thr, D-Leu, Leu, D-Thr, Asn, Thr, D-Leu, D-Leu, X, Asn, D-Leu, D-Ile)
3	NRPS	967071–1036484	Zwittermicin A	7%	(X, Leu, X, Thr, D-Leu, X) + (D-Phe) + (Val, Asp)
4	NRPS–T1PKS hybrid	1047383–1105765			(mal, Ile, Val, D-Phe) + (ohmal)
5	NRPS–T1PKS hybrid	1149253–1299587	O-antigen	10%	(D-Val, Leu, X, X) + (Asp, X, D-X, Leu) + (Ser, X) + (Asp, X ,Asp) + (mal, D-Phe, X, X, X) + (Asp, Pro) + (X, Asp, X, Orn)
6	Terpene	1361172–1380685			
7	NRPS–T1PKS hybrid	1398184–1469526			(X, Leu, Leu, ohmal, pk, X) + (Leu, mal, Trp)
8	Terpene	1628832–1647237			
9	NRPS	1703066–1804498	Zwittermicin A	7%	(X) + (Val, D-Thr, D-Ala, Asp, Phe) + (D-Val, Ser, D-X, X) + (D-Ile, Asp, Leu, Asp, Ile)
10	NRPS	1843907–1909432	Zwittermicin A	7%	(Gly) + (Thr) + (Ala, X) + (ohmal) + (X, X)
11	NRPS	2025378–2065569			(X)
12	Terpene	2079428–2094179			
13	Terpene	2400230–2419564			
14	Lanthipeptide	2599333–2621961			
15	Siderophore	2687915–2699768	Putrebactin / avaroferrin	40%	
16	Lanthipeptide	3135929–3161212	Pinensins	52%	
17	Terpene	3367385–3384768			
18	T3PKS	3412079–3453131			
19	Arylpolyene	3796379–3845920	Flexirubin	100%	
20	Terpene	3985702–4004247			
21	Resorcinol	6391684–6469534	Flexirubin	16%	
22	Aminoglycoside	7080418–6535910			
23	Lassopeptide	7175703–7198253			
24	Terpene	7584305–7605432			
25	NRPS	8277821–8341603	Pellasoren	33%	(X, Thr, Thr, X) + (mal) + (X, Orn, Ala)

[a] BGCs were analyzed by AntiSMASH (https://antismash.secondarymetabolites.org) and PRISM (https://prism.adapsyn.com)

Table S2. Putative ribosomally synthesized and post-translationally modified peptides (RiPPs) in the HK235 genome

Domain	Gene locus	Putative leader-**core** sequence[a]	Most similar known RiPP (leader-**core** sequence)
Lanthipeptide precursor	2614048–2614228	MKKKKIQLNKKLLLKKDKIVTLNPQEQSKLAGG-**VPITWFSSCCVETDEVTCPEGCVWTR**	Unknown
Lanthipeptide precursor	3145197–3145383	MKEQQVSNIKLSIDDLKIDSFVTSIDSEMNMRLAGGLAGQ-**SHPTHTLQTDDEGHLCTTIIC**	Pinensin (MKDNQVTQIKLSIDDLKIDSFVTSIDSEMNMRLAGGLAGQ-**SHPTHTVATDDEGHLCTTIICA)**
Lassopeptide precursor	7188231–7188342	MQEEVLTPGEKKMYQSPELKNLGKIGEVTN-**TTNSGHGNDGGTGPAYNS**<br>MQEEVLTPGEKKMYQSPELKNLGKIGEVTNTT-**NSGHGNDGGTGPAYNS**<br>MQEEVLTPGEKKMYQSPELK-**NLGKIGEVTNTTNSGHGNDGGTGPAYNS**	Unknown

[a] RiPPs were predicted by PRISM (https://prism.adapsyn.com) and RiPPMiner (http://202.54.226.242/~priyesh/rippminer2/new_predictions/index.php)

Table S3. The minimum inhibitory concentration (MIC) of chitinocin against plant pathogens.

Plant pathogen		MIC of chitinocin (ug/ml)
Fungus	*Alternaria brassicicola*	>200
	Botrytis cinerea	200
	Colletotrichum coccodes	50
	Magnaporthe oryzae	>200
	Phytophthora infestans	12.5
Bacterium	*Agrobacterium tumefaciens*	6.3
	Burkholderia cepacia	50
	Clavibacter michiganensis subsp. *michiganensis*	>200
	Erwinia amylovora	50
	Ralstonia solanaceanum	6.3

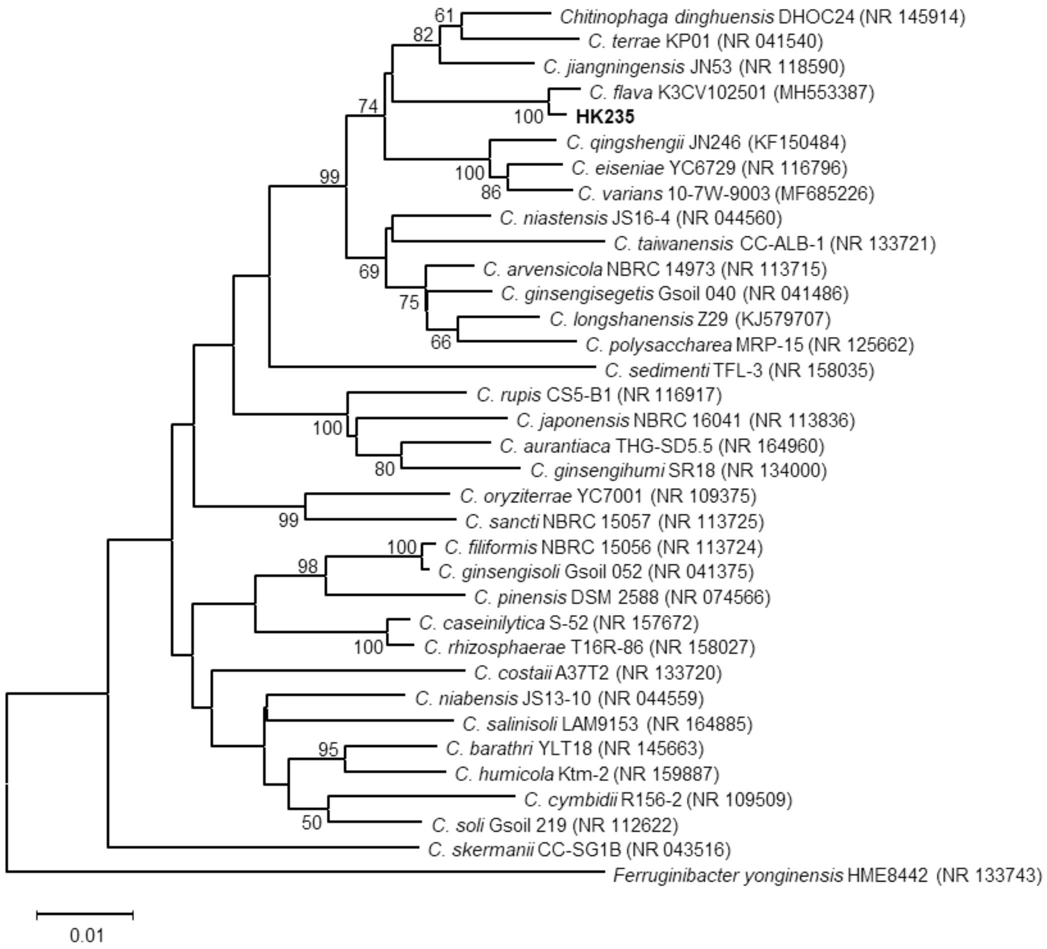

Figure S1. Phylogenetic analysis was performed based on the 16S rRNA gene of HK235 and the other *Chitinophaga* species. The neighbor-joining method was used for this analysis, and numbers at the nodes indicate the levels of bootstrap support (%) by 1000 resampled datasets. NCBI accession numbers of each sequence are in parentheses. Bar, 10 substitution per 1000 nt.

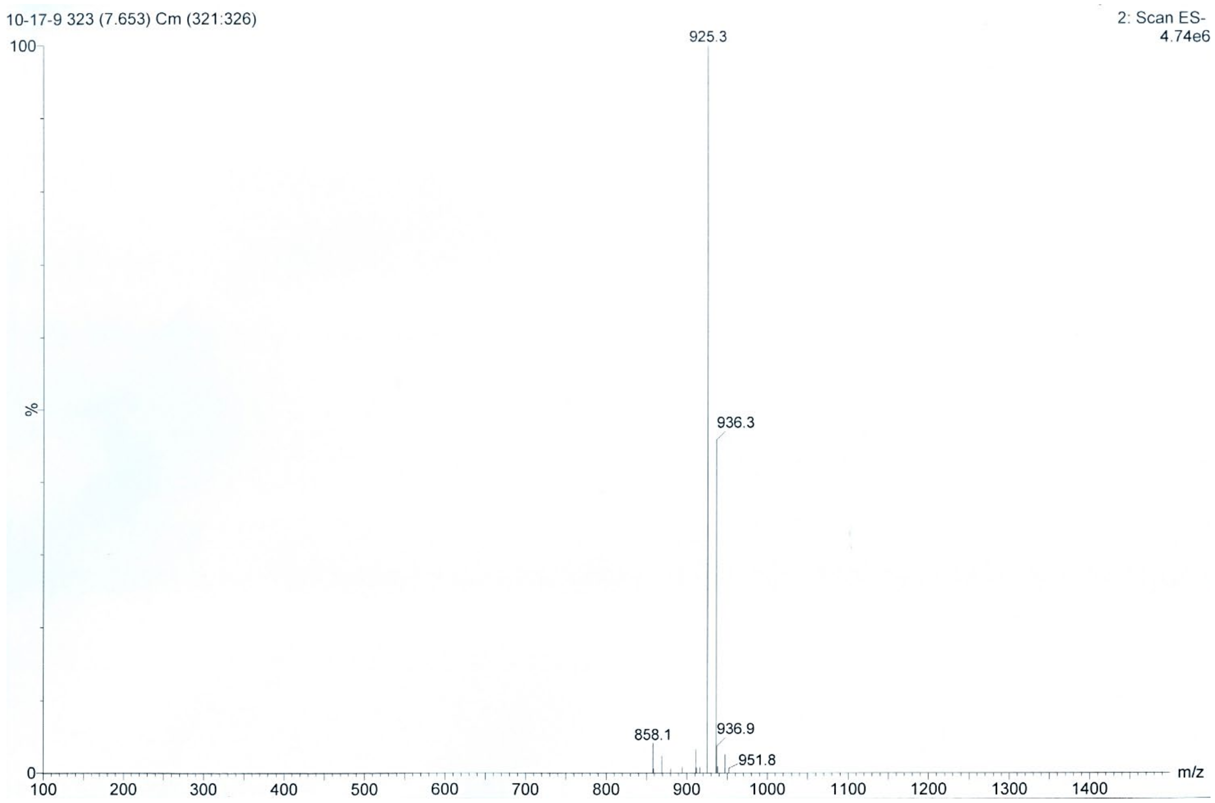

Figure S2. ESI-MS data for chitinocin (MW 1852). Double-charged ion of m/z 925 $[M - 2H]^{2-}$ was observed.

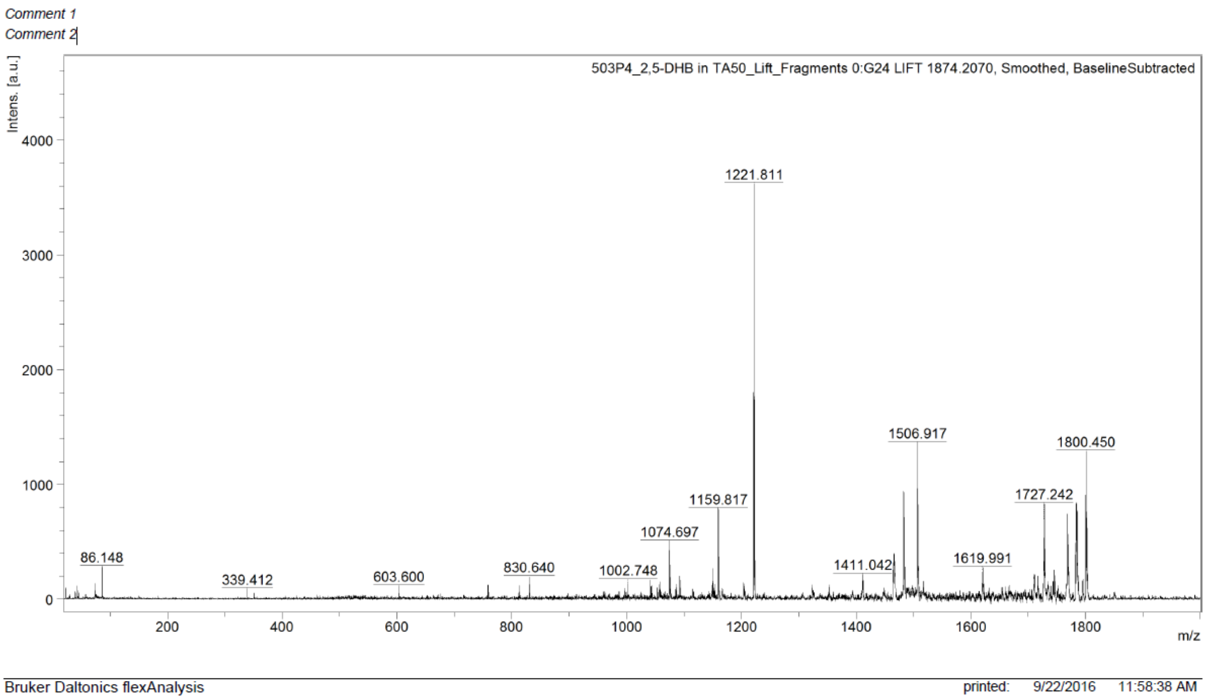

Figure S3. MALDI MS/MS data for chitinocin (MW 1852).

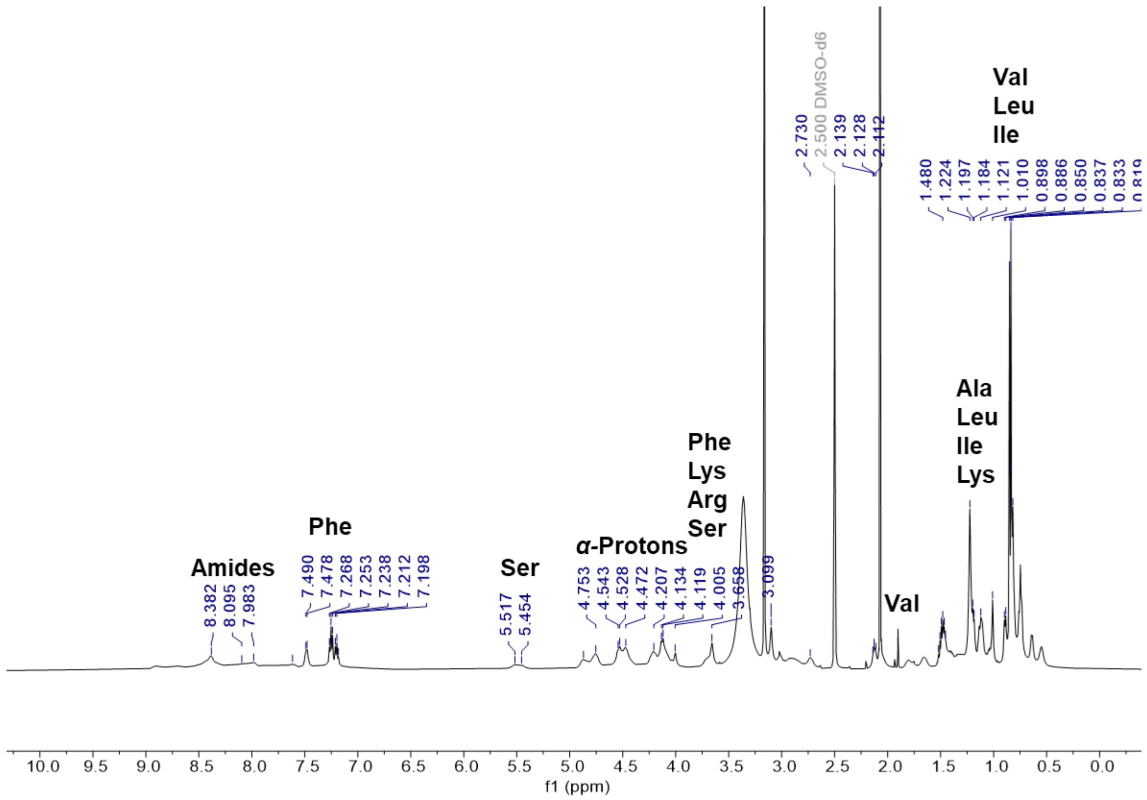

Figure S4. ^{1}H-NMR data for chitinocin.

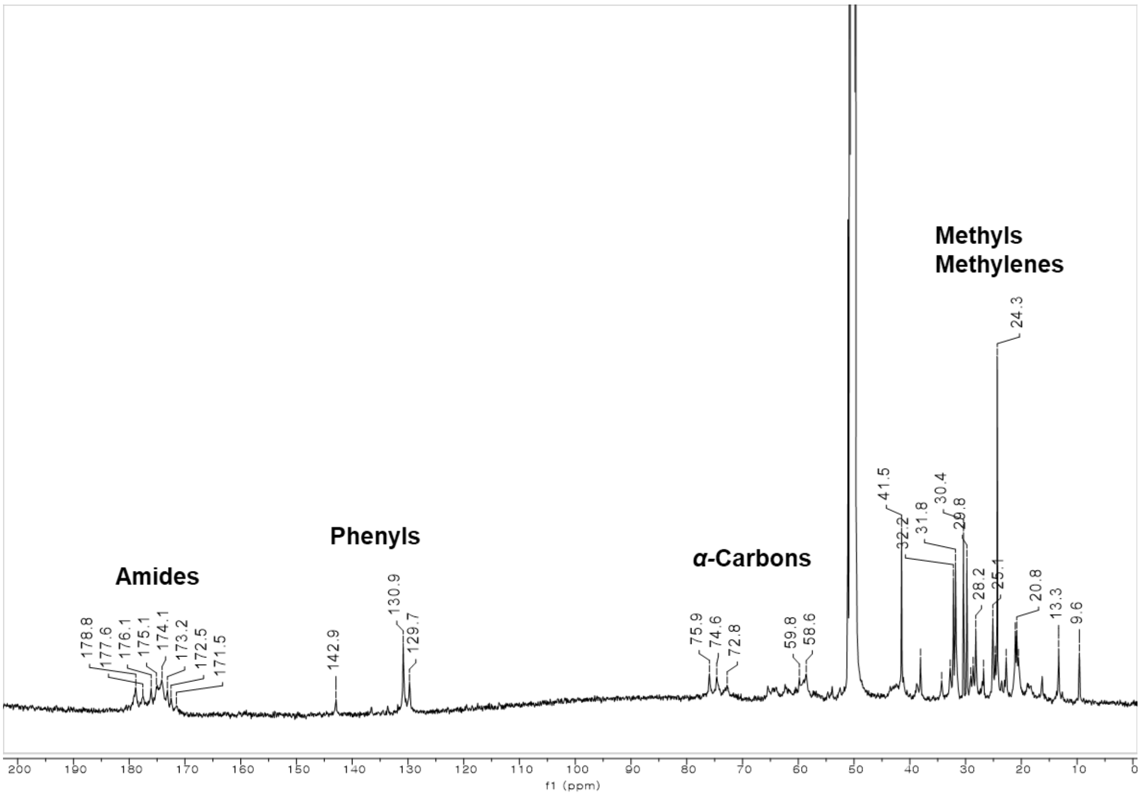

Figure S5. ^{13}C-NMR data for chitinocin.

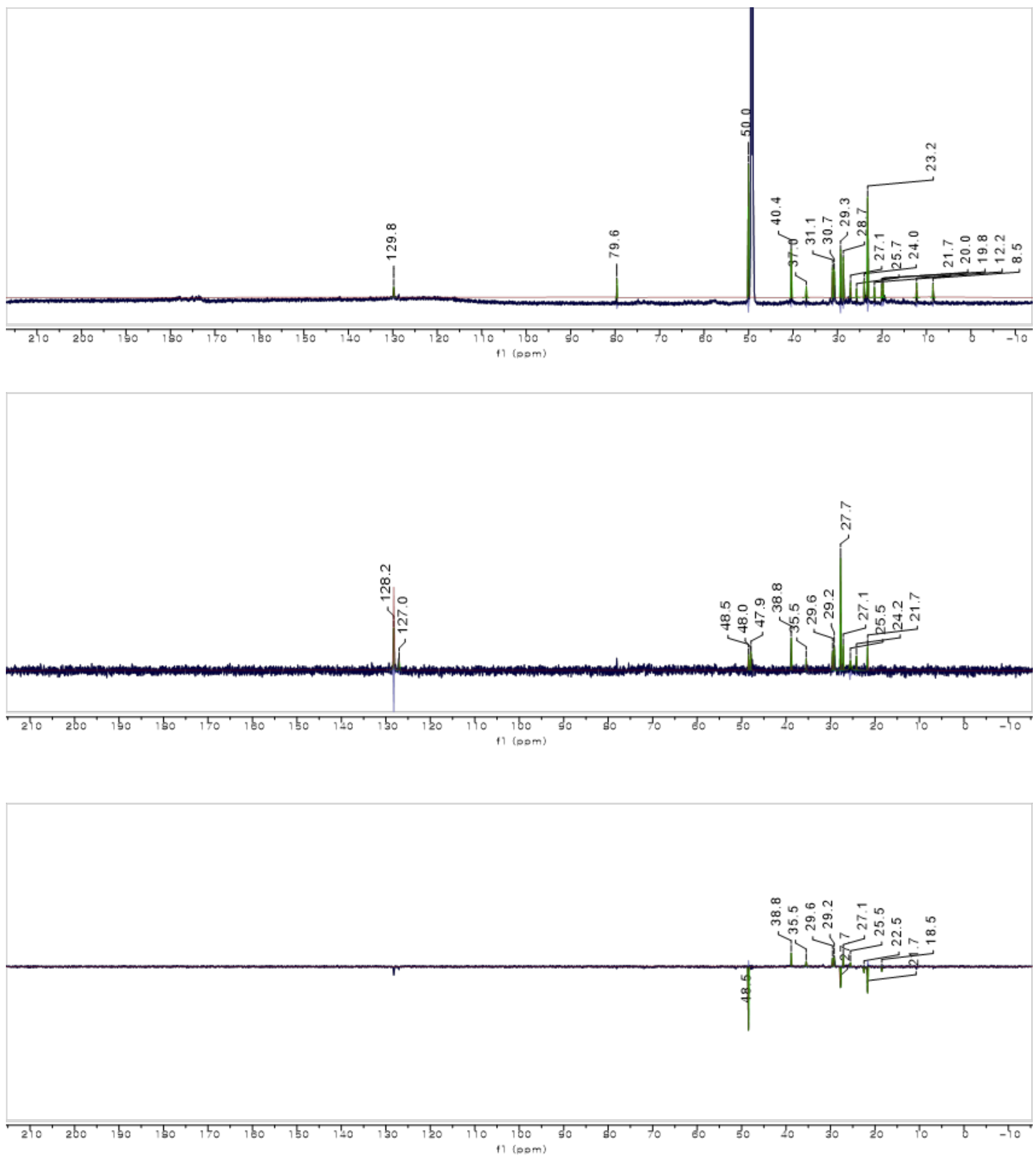

Figure S6. DEPT NMR data for chitinocin. DEPT 45 (top), 90 (middle), and 135 (bottom).

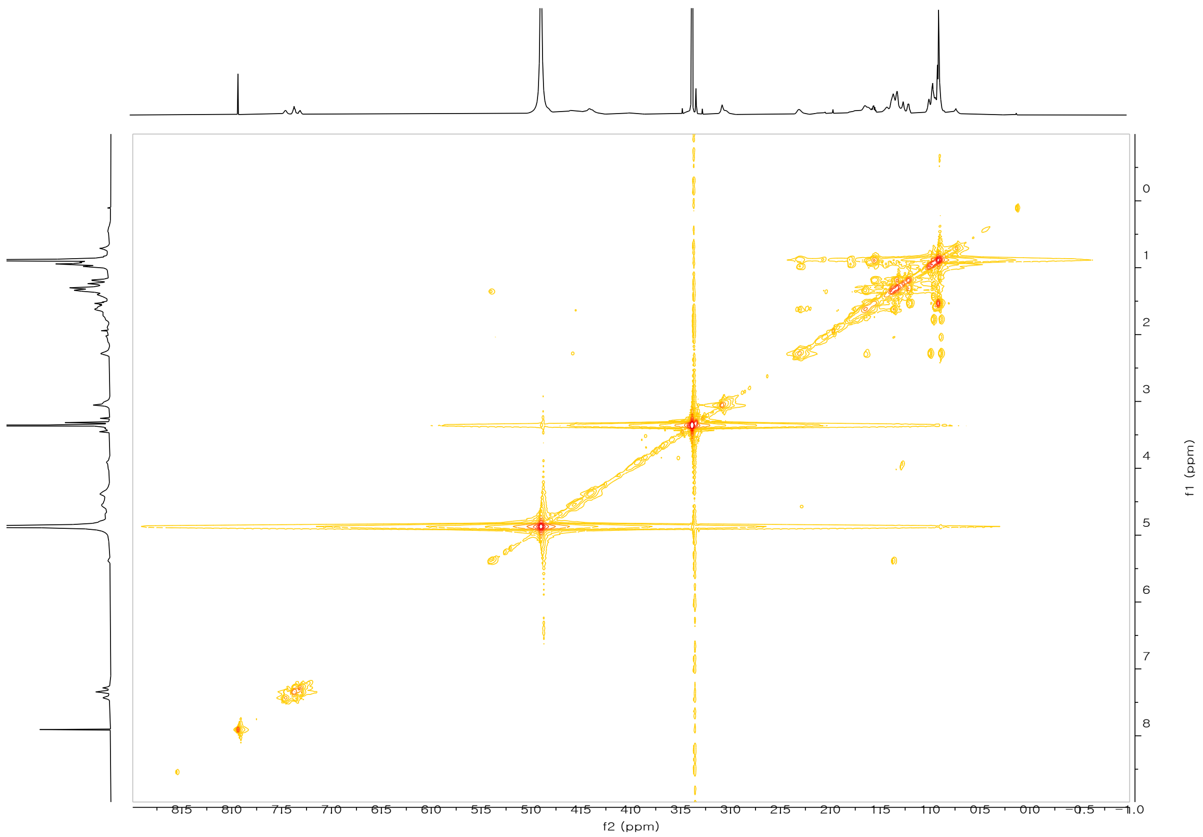

Figure S7. ^{1}H–^{1}H COSY NMR data for chitinocin.

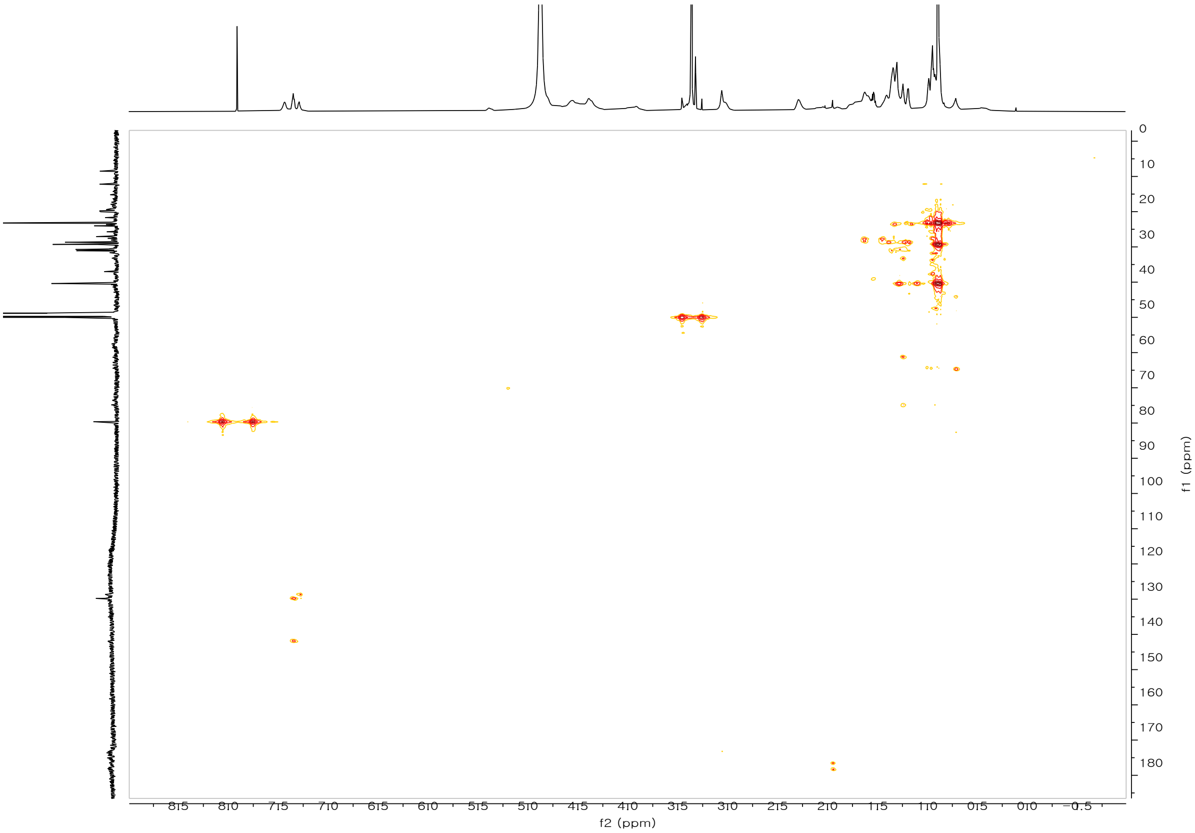

Figure S8. HSQC NMR data for chitinocin.

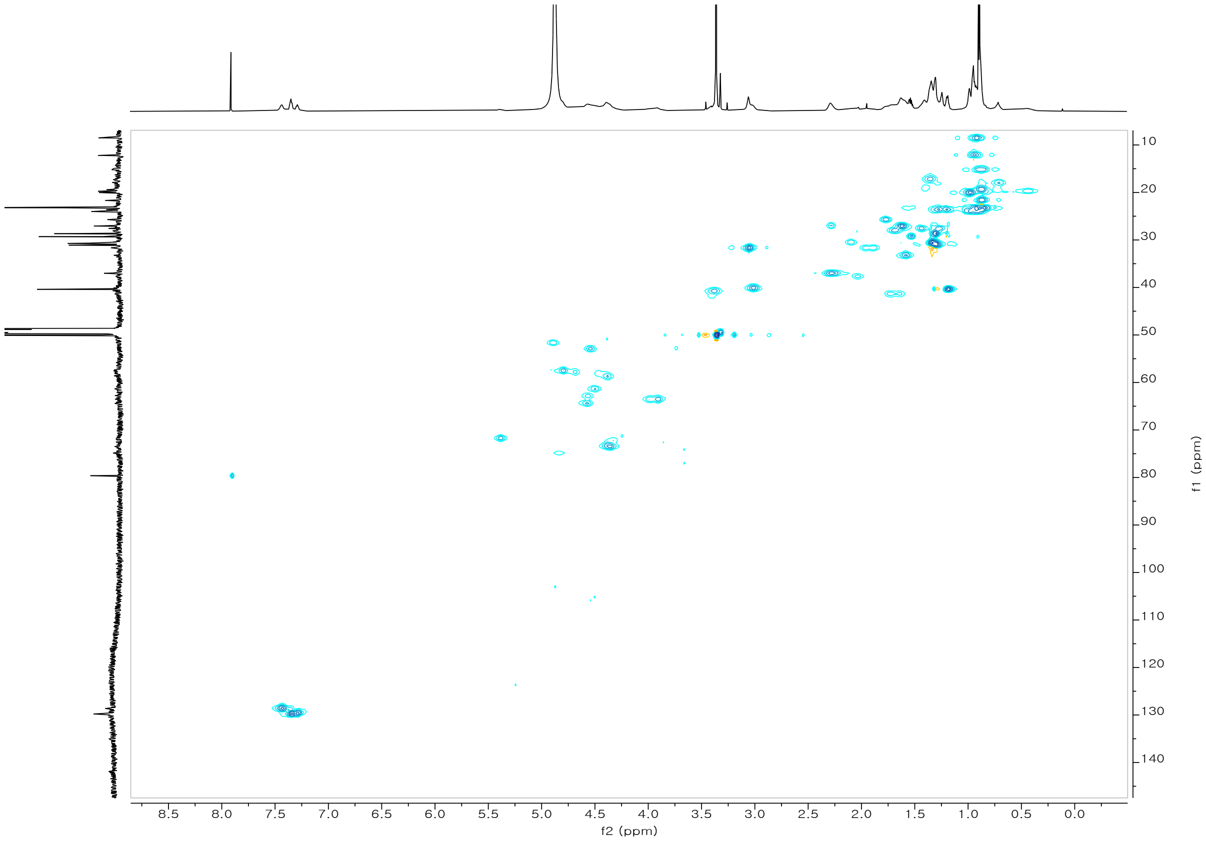

Figure S9. HMBC NMR data for chitinocin.

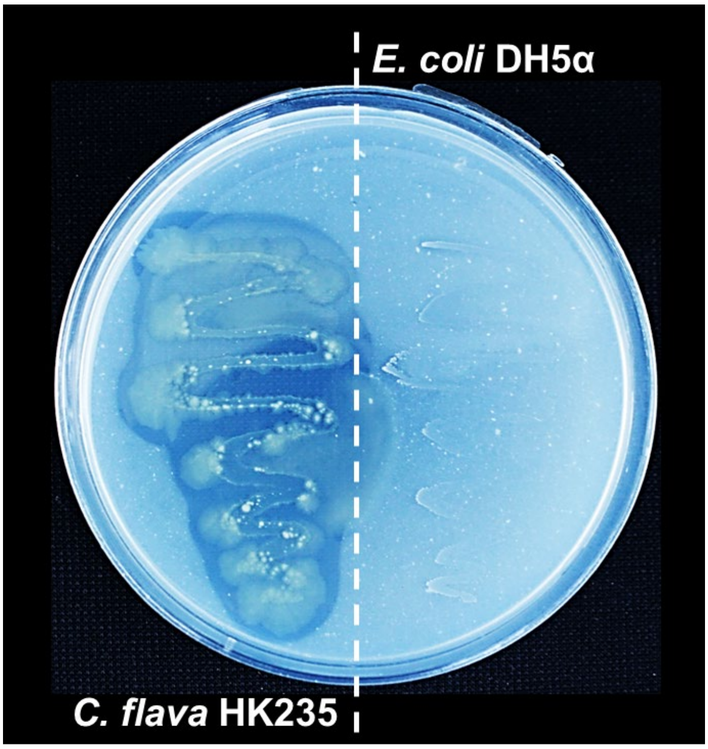

Figure S10. Chitinase activity of the HK235 strain. The clear zone was observed from HK235 grown on colloidal chitin medium 4 dpi at 25 ºC for 4 days.

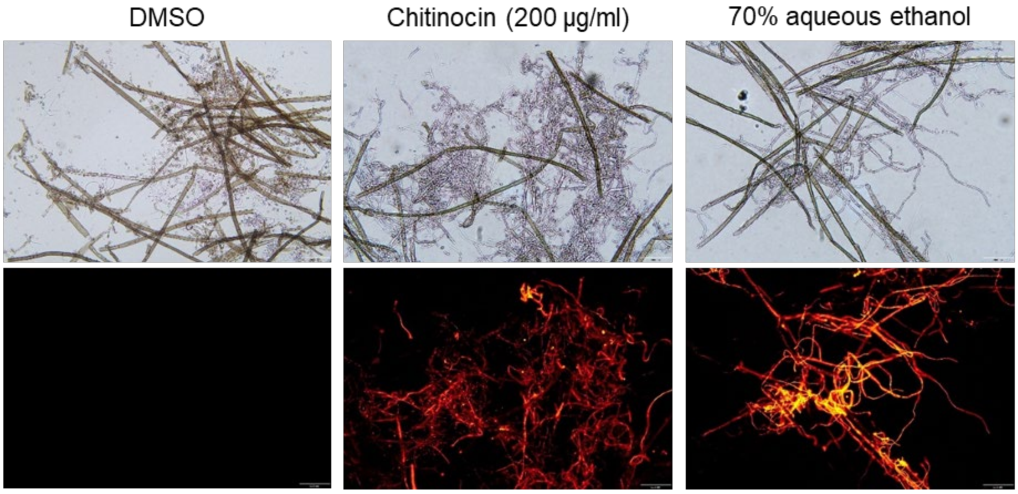

Figure S11. Effects of chitinocin on cell permeability. *B. cinerea* mycelia and conidia were treated by chitinocin for 24 h at 20 °C, and then stained with 2 μM propidium iodide. As a negative and positive control, 1% DMSO and 70% ethanol were used, respectively. The top image was obtained from regular observation, and the bottom image was obtained from fluorescent observation. Scale bars, 50 μm.
